# Glances at the Hospitals

**Published:** 1904-08-20

**Authors:** 


### GLANCES AT THE HOSPITALS.

### DUNDEE ROYAL INFIRMARY.

The total income amounted to £11,775, and the total expenditure to £12,311, showing a deficiency in the income of £536. The number of in-patients treated during the year was 3,500, and the daily average number resident was 232. The average residence of each patient was about 24½ days, and the death-rate was 8·14 per cent.; but 78 patients died within 48 hours of admission; and if these be omitted the death-rate would fall to 5·81 per cent. The cost per bed occupied was £50 14s. 7d. The average daily number of nurses on duty was 59, and it would seem that 374 applications were received from candidates for admission to the nursing staff. The training received at this infirmary is believed to be thoroughly good, and it does not stop with instruction in nursing, but includes training in such cookery as is required by the sick. A library has been opened for the staff, and we are not surprised to hear that it has been found a great source of pleasure and benefit. The medical and surgical statistics are among the best and clearest we have met with. The tables show the number of cases, the results of treatment, the duration of treatment, and there is also a column for remarks. We notice that 33 cases of diphtheria were treated during the year, and of these 24 recovered and 9 died. There were 13 cases of enteric fever and all recovered, the average duration being 54 days. Of 96 cases of pneumonia 20 died. From the anæsthetic table we learn that chloroform was given 1,339 times, ether 25, chloroform and ether 26, A.C.E. mixture 12, nitrous oxide 21, and ethyl chloride 38 times.

### ROYAL COUNTY HOSPITAL AT WINCHESTER.

The ordinary income was £5,341 and the ordinary expenditure was £6,109, showing a deficit of about £768 on the year's working. It should be chronicled that Mr. T. Goddard Williams gave the sum of £1,000 for the purpose of improving and refitting the operating theatre. The committee state that an increased income must be obtained or the hospital must curtail the beneficent work it has hitherto done. The daily average number of patients resident was 79, and the average duration of residence was 29½ days, which is rather high. At the beginning of the year there were 75 patients in residence and 889 were admitted during the year, making a total of 964. Of these 371 recovered, 267 improved greatly, 137 improved somewhat, 72 were discharged for various reasons, 41 died, and 76 remained under treatment. The medical and surgical tables are practically useless as they merely state that a certain number of patients suffered from certain diseases and tell us nothing as to the results. Such tables are not worth the trouble entailed by their compilation. There is no anæsthetic table at all, and we failed to find any mortality statistics. A private nursing institution is attached to the hospital. The charge for nurses in ordinary cases is 30s. a week, and for mental or infectious cases 35s. The estimated profits of this department were £507 during the year.

### THE CHESTERFIELD HOSPITAL.

The total income was £5,002, and it is very pleasing to be able to state the workpeople's own contributions amounted to £2,149. The ordinary expenditure amounted to £4,170 and the extraordinary expenditure to £1,318. The latter sum has been well spent on the operating theatre, nurses' accommodation, alterations to the hot water system, electric lighting, etc. It is worthy of record that Mr. Eastwood gave a donation of £1,000 to the funds, and it is proposed to spend £800 in enlarging the out-patients' department. The governors point out to the inhabitants of the district that they are at present indebted to the Bank to the amount of £2,084, and that to keep the hospital going with the full number of beds in use will need an income of £6,000 a year. The cost per bed occupied was £68 1s., the cost per day per person was nearly 2s. 7d., and the cost per day for food was a trifle over 9d. At the beginning of the year there were 47 patients in residence, and 533 were admitted, making a total of 590 under treatment, and being 89 more than in the previous year. Of this total 362 were discharged cured, 97 were discharged relieved, 19 were discharged for various reasons, 43 died, and 59 remained in the wards. The average number of in-patients in daily residence was 55, and the average duration of treatment was 35 days, which we look upon as very high. We are told that 291 operations were performed; and there is a statement of the cause of death in all cases dying during the year; but beyond these there is a total absence of medical or surgical statistics, a fact which does not reflect very creditably on the medical staff, and is more remarkable inasmuch as the clerical part of the report is fairly well done.

### THE KENT AND CANTERBURY HOSPITAL.

The ordinary income was £3,584 and the ordinary expenditure was £4,555, showing a deficit of £971 on the year's working, which, added to the deficit of the previous year makes a total of £2,200. To meet this the Governors authorised a sale of stock owned by the Hospital, but manifestly this is not a very pleasant expedient, and it is not one that can be practised often to any great extent. The board may study rigid economy in the ordinary expenditure, they may close some of the wards, or they may use greater exertions to increase the annual subscriptions. The latter is the best course. Turning to the details of the income, we notice that the general subscriptions amount to £1,212, which is a fair average for the size of the hospital; but the workpeople's own subscriptions amount to only £148, and although this is considerably higher than on the previous year, it is still much under what might reasonably be expected in the district. Another point is that nothing appears under the heading of probationers' fees. What is the cause of this? It is well known that many hospitals have far more applications from probationers than they can possibly deal with; and there can hardly be any reason why a first-rate training should not be procured in the Canterbury Hospital, which has a daily average of 74 patients. Many structural improvements are necessary, and it has been decided to carry these out at an estimated expense of £7,000, for which the Governors appeal to the public, and it is to be hoped that the appeal will be liberally responded to. On the first of January the hospital contained 74 patients, and 776 were admitted during the year, making a total of 850. Of these 693 were discharged either cured or relieved, 13 not relieved, 8 for other reasons, 58 died, and 78 remained in the wards. The daily average number resident was 73, and the average duration of treatment was 30 days. We do not find any statement relative to the cost per bed occupied, or of the cost per head per day. Among the medical diseases treated during the year we notice 11 cases of acute rheumatism with 9 recoveries and 2 improved, and 6 cases of enteric fever with 4 recoveries and 2 deaths. The table of operations gives details of all the operations performed. Ovariotomy 5 times, all being successful, and appendicectomy 3 times with similar results. There were 3 cases of colotomy with 2 deaths. Five operations for the radical cure of hernia, all being successful. Altogether about 300 operations were performed during the year, most of them, doubtless, under an anæsthetic, but there is no table given showing the nature of the agent used.

### RAMSGATE GENERAL HOSPITAL.

The total receipts were £1,102 10s., and the expenditure was £1,087 17s., leaving a balance on the right side of upwards of £14 on the year's working—a creditable condition of affairs for all concerned in the management. The total number of in-patients was 140; and of these 106 were discharged cured, 13 were discharged relieved, 7 died, and 14 remained in the hospital. In addition to the 140 in-patients, 297 casualty patients were treated, and the total number of operations performed was 69. The medical and surgical tables are well arranged, with columns showing those operated on, those cured, relieved, or died, but we miss a table of anæsthetics.

